# Supplementary material for: Impact of imperceptible motion delay in avatar head movement away from a target on preference formation
Source: PLoS One. 2025 Nov 3;20(11):e0328979. doi: 10.1371/journal.pone.0328979 (PMC12582448; doi:10.1371/journal.pone.0328979)
Supplement: S1 Appendix — Pseudocode describing the proposed method of head-movement intervention using motion delay. (DOCX) [file pone.0328979.s001.docx]

**Supporting Information 1**

**Algorithm: Head movement intervention using motion delay**

// Initial state: the head of the avatar is synchronized with the head of the operator, $t$ is the current time point, Array records $\theta_{opr}$ at every time point $t$

$t$ = 0;

$\theta_{avtr}\left( t \right)=\theta_{opr}(t)$;

Array = []

// Execute the code below until the current trial ends

// Calculate angular velocity of yaw motion of the head of the operator

$\dot{\theta}_{opr}\left( t \right)={{(\theta}_{opr}\left( t \right)-\theta_{opr}(t-\Delta t))}/{\Delta t}$;

// Condition of motion delay not exists

while *motion delay deactivation* do:

// No motion delay exists when operator’s head is stationary or moving toward the target

if $\left| \dot{\theta}_{opr}\left( t \right) \right|\leq Thr\_vel$:

$\theta_{avtr}\left( t \right)=\theta_{opr}(t)$;

else:

// The head of the operator is moving opposite to target

if $\dot{\theta}_{opr}\left( t \right)/\theta_{target}<0$:

// Motion delay is activated

*motion delay activation*;

// Array records $\theta_{opr}$ at every time point $t$ after being initialized as empty

Array = [];

Array[0] = $\theta_{opr}(t)$;

// $\theta_{avtr}$ will be maintained as $\theta_{avtr\_delay}$ in the period of motion delay

$\theta_{avtr\_delay}=\theta_{opr}\left( t \right)$;

$\theta_{avtr}\left( t \right)=\theta_{avtr\_delay}$;

// Condition of motion delay exists

while *motion delay activation* do:

// $\theta_{avtr}$ is maintained as $\theta_{avtr\_delay}$ in the period of motion delay

while the period of motion delay not ends:

$\theta_{avtr}\left( t \right)=\theta_{avtr\_delay}$;

// After the period of motion delay, the head of avatar will follow the trajectory of head movement of the operator. This process ends when the head of avatar is synchronized with the head of the operator

// When the head of avatar is not synchronized with the head of operator

if $\left| \theta_{avtr}\left( t \right)-\theta_{opr}(t) \right|>difference$:

// The head of avatar follows the trajectory of the head of operator

$\theta_{avtr}\left( t \right)=$ Array[0];

for i from 1 to length(Array)-1 do:

Array[i-1] = Array[i];

end for

else:

// When the head of avatar is synchronized with the head of operator

*motion delay deactivation*;

$\theta_{avtr}\left( t \right)=\theta_{opr}(t)$;
