# Supplementary material for: Impact of imperceptible motion delay in avatar head movement away from a target on preference formation
Source: PLoS One. 2025 Nov 3;20(11):e0328979. doi: 10.1371/journal.pone.0328979 (PMC12582448; doi:10.1371/journal.pone.0328979)
Supplement: S3 Appendix — The description and an example of the survey forms for rating the attractiveness of computer-generated faces are listed in this file. (DOCX) [file pone.0328979.s003.docx]

**Supporting Information 3**

**Survey forms for rating attractiveness of computer-generated faces**

In the Rating Phase of the preference formation influence experiment, participants first rated the attractiveness of 40 male European computer-generated faces, followed by 40 female European computer-generated faces.

The survey was conducted using Google Forms, where each face image was presented on a separate page. Participants rated the attractiveness of each face on a scale ranging from 1 to 7, with 1 being "very unattractive" and 7 being "very attractive."

After rating the attractiveness of a face, participants clicked the “next” button to proceed to the next face image, continuing this process until all images had been rated.

As an example, the first male European face image to be rated by participants is shown below.


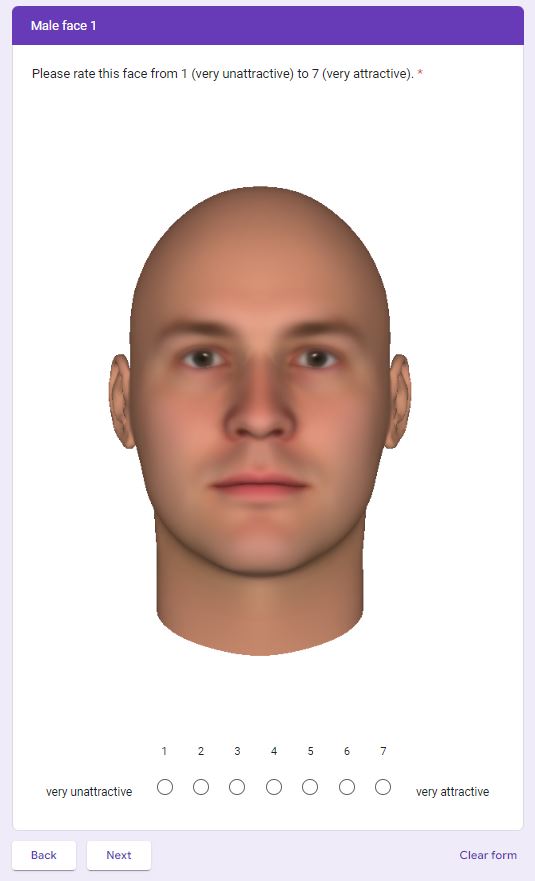


**The first male European face image to be rated by participants**
