# Supplementary material for: Impact of imperceptible motion delay in avatar head movement away from a target on preference formation
Source: PLoS One. 2025 Nov 3;20(11):e0328979. doi: 10.1371/journal.pone.0328979 (PMC12582448; doi:10.1371/journal.pone.0328979)
Supplement: S4 Appendix — Python implementation for calculating Cohen’s d, which is a measure of effect size. (DOCX) [file pone.0328979.s004.docx]

**Python code for calculating Cohen’s d**

import numpy as np

def cohens_d(group1, group2):

# Calculating means of the two groups

mean1, mean2 = np.mean(group1), np.mean(group2)

# Calculating pooled standard deviation

std1, std2 = np.std(group1, ddof=1), np.std(group2, ddof=1)

n1, n2 = len(group1), len(group2)

pooled_std = np.sqrt(((n1 - 1) * std1 ** 2 + (n2 - 1) * std2 ** 2) / (n1 + n2 - 2))

# Calculating Cohen's d

d = (mean1 - mean2) / pooled_std

return d
