# Supplementary material for: Impact of imperceptible motion delay in avatar head movement away from a target on preference formation
Source: PLoS One. 2025 Nov 3;20(11):e0328979. doi: 10.1371/journal.pone.0328979 (PMC12582448; doi:10.1371/journal.pone.0328979)
Supplement: Attachment — Submitted filename: Response to Reviewers.docx [file pone.0328979.s005.docx]

**Response to Editor:**

**Comment 1**

If applicable, we recommend that you deposit your laboratory protocols in protocols.io to enhance the reproducibility of your results. Protocols.io assigns your protocol its own identifier (DOI) so that it can be cited independently in the future. For instructions see: https://journals.plos.org/plosone/s/submission-guidelines#loc-laboratory-protocols. Additionally, PLOS ONE offers an option for publishing peer-reviewed Lab Protocol articles, which describe protocols hosted on protocols.io. Read more information on sharing protocols at https://plos.org/protocols?utm_medium=editorial-email&utm_source=authorletters&utm_campaign=protocols.

**Answer**

Thank you for your suggestion regarding the deposition of laboratory protocols on protocols.io. We appreciate the opportunity to improve the reproducibility of our study. However, we believe that the experimental procedures and system architecture have already been described in sufficient detail in the main text of our manuscript. Therefore, we would prefer to proceed without submitting a separate protocol to protocols.io at this time. Please let us know if further clarification is required.

**Comment 2**

When submitting your revision, we need you to address these additional requirements.

1. Please ensure that your manuscript meets PLOS ONE's style requirements, including those for file naming.

**Answer**

Based on this requirement, we downloaded the LaTeX template from https://journals.plos.org/plosone/s/latex. Our manuscript has been formatted accordingly using this template, and the naming conventions for the figures and appendices have also been adjusted to comply with the submission system's requirements.

**Comment 3**

2. Thank you for stating the following in the Acknowledgments Section of your manuscript:

[This work was supported by the Japan Science and Technology Agency Moonshot Research and Development Program under Grant JPMJMS2011.]

We note that you have provided funding information that is not currently declared in your Funding Statement. However, funding information should not appear in the Acknowledgments section or other areas of your manuscript. We will only publish funding information present in the Funding Statement section of the online submission form.

Please remove any funding-related text from the manuscript and let us know how you would like to update your Funding Statement. Currently, your Funding Statement reads as follows:

[This work was funded by Japan Science and Technology Agency Moonshot Research and Development Program (grant #JPMJMS2011). The URL is "https://www.jst.go.jp/moonshot/en/index.html". YN and HI were the authors who received award from the mentioned funder. No sponsors or funders had a role in the study design, data collection and analysis, decision to publish, or preparation of the manuscript.]

Please include your amended statements within your cover letter; we will change the online submission form on your behalf.

**Answer**

Based on this suggestion, we removed “This work was supported by the Japan Science and Technology Agency Moonshot Research and Development Program under Grant JPMJMS2011” from the Acknowledgments section and revised it as “We thank the two reviewers for their excellent feedback, which has improved this manuscript.”

**Comment 4**

3. When completing the data availability statement of the submission form, you indicated that you will make your data available on acceptance. We strongly recommend all authors decide on a data sharing plan before acceptance, as the process can be lengthy and hold up publication timelines. Please note that, though access restrictions are acceptable now, your entire data will need to be made freely accessible if your manuscript is accepted for publication. This policy applies to all data except where public deposition would breach compliance with the protocol approved by your research ethics board. If you are unable to adhere to our open data policy, please kindly revise your statement to explain your reasoning and we will seek the editor's input on an exemption. Please be assured that, once you have provided your new statement, the assessment of your exemption will not hold up the peer review process.

**Answer**

Based on this suggestion, we uploaded the dataset to Figshare. It is available at the following URL: https://figshare.com/s/91351a3e40c26e35ae9f.

**Comment 5**

4. We are unable to open your Figure file [Fig3.eps]. Please kindly revise as necessary and re-upload.

**Answer**

We appreciate your bringing this issue to our attention. We have recreated Fig3.eps and confirmed that it can now be opened without any issues. The corrected version of Fig3.eps has been re-uploaded.

**Comment 6**

5. Please review your reference list to ensure that it is complete and correct. If you have cited papers that have been retracted, please include the rationale for doing so in the manuscript text, or remove these references and replace them with relevant current references. Any changes to the reference list should be mentioned in the rebuttal letter that accompanies your revised manuscript. If you need to cite a retracted article, indicate the article’s retracted status in the References list and also include a citation and full reference for the retraction notice.

**Answer**

Thank you for bringing this issue to our attention. We have reviewed all the references cited in our manuscript and confirmed that none have been retracted.

**Response to Reviewer: 1**

**Comment 1**

1. The introduction gives a general overview but does not explain the problem addressed or why the proposed method is novel in comparison to earlier work.

**Answer**

Based on this suggestion, we compared the present study with our previous work to highlight the novelty of the proposed method. Two key reasons are outlined in the introduction. First, our previous approach required the calibration of three parameters to ensure that the manipulation remained imperceptible to the operators. In contrast, the current method requires only a single parameter, which significantly simplifies the implementation of the avatar system. Second, unlike traditional approaches that aim to minimize motion delay because of its negative impact on user experience, our method intentionally leverages subtle motion delay as a tool to achieve cognitive influence.

**Introduction, Paragraph 4 is revised as follows:**

**“**However, a limitation of our previous study was the need to determine three parameters: the imperceptible speed of the avatar's head when the operator’s head was stationary, moving toward the target, or moving away from the target. The measurement of these parameters is complex, which may hinder the practical application of the proposed method. However, motion delay---an inherent characteristic of teleoperation systems\cite{farajiparvar2020brief} that disrupts operators' intentional movements---is often regarded as a negative factor impacting user experience in avatar operations\cite{claypool2006latency,kim2021impact}, particularly when the delay is significant. Although motion delay has traditionally been viewed as a negative factor in teleoperation systems, it is worth investigating whether such delays---particularly when subtle and imperceptible---can be positively applied. For example, motion delay may be leveraged to influence human cognition, such as shaping perceptual judgments or guiding preference formation during observational tasks.”

**Introduction, Paragraph 5 is revised as follows:**

**“**In this study, we proposed a method that leverages motion delay to prevent the operator from looking away from a target, thereby facilitating the development of a preference for that target. Compared to our previous study---which required a complex measurement of three separate parameters to ensure the imperceptibility of head movement manipulation---the current approach simplifies the process by using only a single parameter: motion delay. Notably, although motion delay is typically considered a negative factor that should be minimized in teleoperation systems, our approach utilizes it constructively. Specifically, this delay negatively affects preference formation toward non-target objects. By introducing a subtle motion delay when the operator attempts to shift their gaze away from the target, we hypothesized that this delay would make it harder to observe the non-target in that direction, thereby enhancing the preference of the operator for the target. We aimed to explore the potential of motion delay as a subtle and effective mechanism for influencing operator behavior. To achieve this, we developed a system capable of introducing motion delays, specifically when the head of the avatar moves away from the target object. In the main experiment, we aimed to intentionally influence preference formation toward computer-generated faces using these imperceptible motion delays. Specific delay values were determined based on the results of a pilot study. Our findings demonstrated that imperceptible motion delays in avatar operations can be used to intentionally influence preference formation.”

**Comment 2**

2. The article does not provide comprehensive descriptions of the steps involved in data collection and signal processing; this should be improved.

**Answer**

Based on this suggestion, we added a subsection “Data collection and processing” in Section “Materials and methods”. We described the collection of data in the first paragraph and subsequently mentioned how to process the data we collected in the second paragraph.

**Materials and methods, Data collection and processing, Paragraph 1 is written as follows:**

**“**In the preference formation influence experiment, operators were required to rotate their head to observe each face image at least five times per trial. For each trial, we recorded the yaw rotation angles of both $Head_{opr}$ and $Head_{avtr}$ at each time step. Additionally, to evaluate the extent to which the operator’s choices aligned with the target faces, we recorded the direction of the target face and the operator’s final selection for each trial.”

**Materials and methods, Data collection and processing, Paragraph 2 is written as follows:**

**“**For each trial, we plotted the yaw rotation angles of both $Head_{opr}$ and $Head_{avtr}$ over time to visualize their movement paths. To ensure data quality, we excluded an operator’s data if they failed to observe each face image at least five times in three or more trials. This criterion was used to ensure sufficient engagement with stimuli. To evaluate how well the operators’ final selections matched the target faces, we counted the number of trials (out of ten) in which each operator selected the target face. These counts were then compared to the expected frequency under a random selection scenario (50\%) using a one-tailed paired-samples t-test to evaluate the impact of the intervention. This analysis was conducted in Python using the function \texttt{scipy.stats.ttest\_rel}. Prior to the t-test, the normality of the data distribution was assessed using the Shapiro--Wilk test, implemented via \texttt{scipy.stats.shapiro}. In addition to assessing statistical significance, we calculated Cohen’s d to evaluate the size of the effect (\nameref{S4_Appendix}).”

**Comment 3**

3. The statistical methods used for data analysis are not explained in detail; it should be explained more thoroughly how the tests were conducted and whether the expectations of the proposed method were met.

**Answer**

Based on this suggestion, we provided a detailed explanation of how the statistical tests were conducted in the second paragraph of the "Data Collection and Processing" subsection. A one-tailed paired-samples t-test and Shapiro–Wilk test were performed using the Python functions. Cohen’s d was also calculated with Python, and the corresponding code is provided in Appendix S4.

**Materials and methods, Data collection and processing, Paragraph 1 is written as follows:**

**“**For each trial, we plotted the yaw rotation angles of both $Head_{opr}$ and $Head_{avtr}$ over time to visualize their movement paths. To ensure data quality, we excluded an operator’s data if they failed to observe each face image at least five times in three or more trials. This criterion was used to ensure sufficient engagement with stimuli. To evaluate how well the operators’ final selections matched the target faces, we counted the number of trials (out of ten) in which each operator selected the target face. These counts were then compared to the expected frequency under a random selection scenario (50\%) using a one-tailed paired-samples t-test to evaluate the impact of the intervention. This analysis was conducted in Python using the function \texttt{scipy.stats.ttest\_rel}. Prior to the t-test, the normality of the data distribution was assessed using the Shapiro--Wilk test, implemented via \texttt{scipy.stats.shapiro}. In addition to assessing statistical significance, we calculated Cohen’s d to evaluate the size of the effect (\nameref{S4_Appendix}).”

We explained whether the expectations of the proposed method were met in the “Results” section.

**“Results” section is revised as follows:**

**“**The results of the preference formation experiment are shown in Fig.~\ref{fig3}. On average, participants selected the target face as their preferred option 59.2\% of the time, exceeding the 50\% chance level. The Shapiro--Wilk test confirmed that the data did not significantly deviate from normality, \emph{p} = 0.393. A one-tailed paired-samples t-test showed that participants selected the target face significantly more often than by chance (\emph{M} = 59.2\%, \emph{SD} = 1.56), \emph{t}(23) = 2.88, \emph{p} = 0.004, Cohen's d = 0.83, indicating a large size effect.”

**Comment 4**

4. The results section clearly presents the findings, but a more thorough discussion of statistical significance and effect sizes is advised.

**Answer**

Information on statistical significance and effect sizes is included in the “Results” section.

**“**The results of the preference formation experiment are shown in Fig.~\ref{fig3}. On average, participants selected the target face as their preferred option 59.2\% of the time, exceeding the 50\% chance level. The Shapiro--Wilk test confirmed that the data did not significantly deviate from normality, \emph{p} = 0.393. A one-tailed paired-samples t-test showed that participants selected the target face significantly more often than by chance (\emph{M} = 59.2\%, \emph{SD} = 1.56), \emph{t}(23) = 2.88, \emph{p} = 0.004, Cohen's d = 0.83, indicating a large size effect.”

**Comment 5**

5. The main text does not adequately explain certain figures. It is advised that the narrative include explicit citations and interpretations for all figures and tables.

**Answer**

Based on this suggestion, we have included explicit citations and interpretations for all three figures.

**Figure 1, mentioned in the first paragraph of Section “Materials and methods”, subsection “Head-movement intervention method using motion delay.”**

　　“We propose a head-movement intervention method that introduces motion delay to subtly prevent avatar head movement, denoted as $Head_{avtr}$, from aligning with operator head movement, denoted as $Head_{opr}$ (Fig~\ref{fig1}). As illustrated in Fig~\ref{fig1}a, the operator wears a head-mounted display and observes objects, including the target object, through a virtual avatar system. Our proposed method introduces a motion delay when the operator attempts to look at a non-target object. Therefore, in the context of observing two objects, no motion delay is introduced when $Head_{opr}$ moves toward the target object or is considered stationary. During this process, the movement of $Head_{avtr}$ was fully synchronized with that of $Head_{opr}$, thereby ensuring ease of operator observation. However, when the operator moves the head away from the target to observe another object, an imperceptible motion delay is applied to the movement of $Head_{avtr}$. This delay temporarily halts the movement of $Head_{avtr}$ for a brief period, resulting in a slight desynchronization between $Head_{opr}$ and $Head_{avtr}$ (Fig~\ref{fig1}b). Once the delay period ends, $Head_{avtr}$ starts following the trajectory of $Head_{opr}$, allowing the operator to perceive its movement as natural (Fig~\ref{fig1}c). This continues until $Head_{avtr}$ and $Head_{opr}$ are fully synchronized (Fig~\ref{fig1}d). As a result, the operator will find it more challenging to observe the nontarget object owing to the motion delay. The algorithm used in our proposed method, which introduces an imperceptible motion delay to intervene with the avatar’s head movement, is detailed in \nameref{S1_Appendix}.”

**Figure 2, mentioned in the first paragraph of Section “Materials and methods”, subsection “Experimental setup.”**

　　“A head-mounted display (HMD; Oculus Quest 2, Oculus VR, Inc.) was used to observe the stimuli in a virtual environment. The operators were required to press one of the two buttons to respond to the stimuli. Additionally, a pair of headphones (SONY WH-1000XM5, Sony Group Corporation) was used to play white noise during the experiments. A photograph of the experimental setup is presented in Fig~\ref{fig2}a, and Fig~\ref{fig2}b shows a schematic of the setup.”

**Figure 3, mentioned in the Section “Results.”**

　　“The results of the preference formation experiment are shown in Fig.~\ref{fig3}. On average, participants selected the target face as their preferred option 59.2\% of the time, exceeding the 50\% chance level. The Shapiro--Wilk test confirmed that the data did not significantly deviate from normality, \emph{p} = 0.393. A one-tailed paired-samples t-test showed that participants selected the target face significantly more often than by chance (\emph{M} = 59.2\%, \emph{SD} = 1.56), \emph{t}(23) = 2.88, \emph{p} = 0.004, Cohen's d = 0.83, indicating a large size effect.”

**Comment 6**

6. The discussion section does not thoroughly examine the ramifications of the findings and is somewhat short. It is necessary to conduct a more thorough examination of how these findings add to the corpus of current knowledge.

**Answer**

Based on this suggestion, we removed the second paragraph of the Discussion section. In its place, we added a new paragraph, which is now the fourth paragraph of the Discussion section, to compare the current study with our previous work. In addition, we highlighted that motion delay can be intentionally utilized as a tool and emphasized the potential of influencing operator cognition through interventions on the avatar's movements rather than direct manipulation of the operator.

**Discussion, Paragraph 4**

　　“In our previous study \cite{preprint}, we imperceptibly guided the head of the avatar toward the target face to facilitate observation, thereby guiding the operators to choose the target face as the preferred face. However, this method requires the complex measurement of three parameters to ensure that the manipulation remains imperceptible, which makes practical application difficult. Therefore, reducing the number of required parameters is beneficial. In the present study, we aimed to achieve a similar effect on preference formation using a simpler approach that relies on only one parameter: motion delay. Additionally, although motion delay is typically regarded as a negative factor in teleoperation systems---something to be minimized---we intentionally utilized it to influence preference formation toward the target objects. Our findings demonstrate the potential for motion delay. To determine which of the two approaches had a stronger effect on preference formation, we conducted an independent samples t-test to compare the means of the two conditions. There was no significant difference between the groups, \emph{t}(50) = -0.553, \emph{p} = 0.583. Therefore, statistical analyses indicated no significant difference between the proposed methods in these two studies, suggesting that their effects on preference formation were comparable. Considered together with our previous research, this study highlights the possibility of influencing operator cognition indirectly through the manipulation of avatar movements, rather than through direct intervention with the operators themselves.”

**Response to Reviewer: 2**

**Comment 1**

1. Fig. 1 should be expanded for references that should be included in the content - all other drawings have one, and the specificity of the presented system even requires that should be presented in this way.

The readability of the course shown in Figure 1 should be improved. In addition, the results shown in Figure 1 have been described too scarcely, it is recommended to extend the description of point Head-movement intervention method using motion delay (page 2), so that the reader can obtain more information.

**Answer**

Based on this suggestion, we have included an explicit citation and added an interpretation of Fig. 1. The details are as follows.

**Figure 1, mentioned in the first paragraph of Section “Materials and methods”, subsection “Head-movement intervention method using motion delay.”**

　　“We propose a head-movement intervention method that introduces motion delay to subtly prevent avatar head movement, denoted as $Head_{avtr}$, from aligning with operator head movement, denoted as $Head_{opr}$ (Fig~\ref{fig1}). As illustrated in Fig~\ref{fig1}a, the operator wears a head-mounted display and observes objects, including the target object, through a virtual avatar system. Our proposed method introduces a motion delay when the operator attempts to look at a non-target object. Therefore, in the context of observing two objects, no motion delay is introduced when $Head_{opr}$ moves toward the target object or is considered stationary. During this process, the movement of $Head_{avtr}$ was fully synchronized with that of $Head_{opr}$, thereby ensuring ease of operator observation. However, when the operator moves the head away from the target to observe another object, an imperceptible motion delay is applied to the movement of $Head_{avtr}$. This delay temporarily halts the movement of $Head_{avtr}$ for a brief period, resulting in a slight desynchronization between $Head_{opr}$ and $Head_{avtr}$ (Fig~\ref{fig1}b). Once the delay period ends, $Head_{avtr}$ starts following the trajectory of $Head_{opr}$, allowing the operator to perceive its movement as natural (Fig~\ref{fig1}c). This continues until $Head_{avtr}$ and $Head_{opr}$ are fully synchronized (Fig~\ref{fig1}d). As a result, the operator will find it more challenging to observe the nontarget object owing to the motion delay. The algorithm used in our proposed method, which introduces an imperceptible motion delay to intervene with the avatar’s head movement, is detailed in \nameref{S1_Appendix}.”

Additionally, we have revised Figure 1 to improve its readability. The superseded and revised Fig. 1 are shown below.

**Fig. 1, before change.**

**Head-movement intervention method using motion delay**

For simplicity, our method applies motion delay only to the yaw rotation of avatar head. theta_{opr}(t) and theta_{avtr}(t) denote the yaw rotation angles of the head of the operator and avatar at a particular time point t, respectively. Similarly, the angular velocities of yaw rotation at time point t are represented as Dot{theta}_{opr}(t) and Dot{theta}_{avtr}(t), corresponding to the operator and avatar, respectively. t_{prev} and t_{next} are two consecutive time points. (a) Illustration of pitch, yaw, and roll rotations. (b) When the operator rotates their head away from the target, an imperceptible motion delay is introduced. The head of the avatar remains stationary during the motion-delay period. (c) Once the motion-delay period ends, the head of the avatar replicates the trajectory of the head movements of the operator. (d) This process concludes when the head of the avatar is synchronized with that of the operator.

**Fig .1, after change.**

**Head-movement intervention method using motion delay.**

For simplicity, our method applies motion delay only to the yaw rotation of avatar head. $\theta_{opr}(t)$ and $\theta_{avtr}(t)$ denote the yaw rotation angles of the head of the operator and avatar at a particular time point $t$, respectively. Similarly, the angular velocities of the yaw rotation at time point $t$ are represented as $\Dot{\theta}_{opr}(t)$ and $\Dot{\theta}_{avtr}(t)$, corresponding to the operator and avatar, respectively. $t_{prev}$ and $t_{next}$ are two consecutive time points, respectively. (a) The operator wears a head-mounted display and observes objects, including the target object, through a virtual avatar system. The pitch, yaw, and roll rotations of the head are shown. (b) When the operator rotates their head away from the target, an imperceptible motion delay is introduced. The head of the avatar remains stationary during the motion-delay period. (c) Once the motion-delay period ends, the head of the avatar replicates the trajectory of the head movements of the operator. (d) This process concludes when the head of the avatar is synchronized with that of the operator.

**Comment 2**

2. Expand the text of the manuscript (or the introduction or conclusion) with specific results in the world and in Europe, - increase the quality of the work by listing the results of publications of researchers and experts working in this field registered in world databases. These are: Analysis of the possibilities of tire-defect inspection based on unsupervised learning and deep learning (21, DOI 10.3390/s21217073), Collaborative assembly task realization using selected type of a human-robot interaction (DOI 10.1016/j.trpro.2019.07.078), Implementation of predictive models in industrial machines with proposed automatic adaptation algorithm (DOI 10.3390/app12041853) and Solutions to the characteristic equation for industrial robot's elliptic trajectories (DOI10.17559/TV-20150114112458) thanks.

The presented literature is accurate, but most of the indicated items are from one country (Japan), it is recommended to extend it by several additional foreign items.

**Answer**

Based on this suggestion, we cited three European studies in Paragraph 1 of the introduction section. The three papers are “A long distance telesurgical demonstration on robotic surgery phantoms over 5G,” “Virtual reality-based dynamic scene recreation and robot teleoperation for hazardous environments,” and “icub3 avatar system: Enabling remote fully immersive embodiment of humanoid robots.” In addition to these papers, we cited one more paper from France, that is, “Impact of communication delay and temporal sensitivity on perceived workload and teleoperation performance.” This study is cited in Paragraph 4 of the introduction.

Thank you for your suggestion to cite the paper:
 *"* *Analysis of the possibilities of tire-defect inspection based on unsupervised learning and deep learning "* (<https://doi.org/10.3390/s21217073>).
After carefully reviewing the content of the paper, we have determined that it does not have direct relevance to our current work, which focuses on a teleoperated avatar system incorporating imperceptible manipulations to influence the operator’s cognition. The referenced paper primarily describes the development of a tire inspection system for the tire industry, where abnormalities are addressed using unsupervised and deep learning techniques, which are fundamentally different from the scope of our study.
Therefore, we respectfully decline to include this citation as it does not provide meaningful context or support for the arguments presented in our manuscript.

Thank you for your suggestion to cite the paper:

*"Collaborative assembly task realization using selected type of a human-robot interaction"* (<https://doi.org/10.1016/j.trpro.2019.07.078>).

After carefully reviewing the content of the paper, we have determined that it does not have direct relevance to our current work, which focuses on a teleoperated avatar system incorporating imperceptible manipulations to influence the operator’s cognition. The referenced study primarily addressed collaborative assembly tasks involving physical co-presence and direct human-robot interaction paradigms, which are fundamentally different from the scope of our study.

Therefore, we respectfully decline to include this citation, as it would not provide meaningful context or support for the arguments presented in our manuscript.

Thank you for your suggestion to cite the paper:
 *"Implementation of Predictive Models in Industrial Machines with Proposed Automatic Adaptation Algorithm"* (<https://doi.org/10.3390/app12041853>).
After carefully reviewing the content of the paper, we have determined that it does not have direct relevance to our current work, which focuses on a teleoperated avatar system incorporating imperceptible manipulations to influence the operator’s cognition. The referenced paper primarily describes the issue of using predictive models of NAR neural networks to predict the course of certain quantities, which may indicate a problem with industrial machines or their major failures, which are fundamentally different from the scope of our study.
Therefore, we respectfully decline to include this citation, as it would not provide meaningful context or support for the arguments presented in our manuscript.

Thank you for your suggestion to cite the paper:

*"Solutions to the characteristic equation for industrial robot's elliptic trajectories"* (<https://doi.org/10.17559/TV-20150114112458>).

After carefully reviewing the content of the paper, we have determined that it does not have direct relevance to our current work, which focuses on a teleoperated avatar system incorporating imperceptible manipulations to influence the operator’s cognition. The referenced paper primarily describes a radical-free analytical method for linear transformations in industrial robots, enabling efficient application in broader engineering contexts through the set-theoretic and algebraic classification of automorphisms.

Therefore, we respectfully decline to include this citation, as it would not provide meaningful context or support for the arguments presented in our manuscript.

**Introduction, Paragraph 1, after revision:**

　　“Avatars, which represent operators in specific environments\cite{castronova2003theory}, are widely used in remote manipulation scenarios, such as online education\cite{petrakou2010interacting,mkrttchian2020avatar,hu2023using} and virtual social communications\cite{teng2023avatar,kobayashi2021avatar,luo2023effect}. They are also earmarked for advanced applications, including remote surgery\cite{morohashi2022social,moustris2023long} and exploration in hazardous environments\cite{szczurek2023multimodal,bavelos2025virtual}. Functioning as extensions of the human body\cite{slater2010first,maister2015changing,rosa2019player}, avatars offer high operability and the ability to overcome physical distances, while ensuring operator safety\cite{szczurek2023multimodal,dafarra2024icub3}. Consequently, their importance has grown significantly, particularly in scenarios involving teleoperation tasks\cite{freeman2021body,miao2022emerging}.”

**Introduction, Paragraph 4, after revision:**

　　“However, a limitation of our previous study was the need to determine three parameters: the imperceptible speed of the avatar's head when the operator’s head was stationary, moving toward the target, or moving away from the target. The measurement of these parameters is complex, which may hinder the practical application of the proposed method. However, motion delay---an inherent characteristic of teleoperation systems\cite{farajiparvar2020brief} that disrupts operators' intentional movements---is often regarded as a negative factor impacting user experience in avatar operations\cite{claypool2006latency,kim2021impact}, particularly when the delay is significant. Although motion delay has traditionally been viewed as a negative factor in teleoperation systems, it is worth investigating whether such delays---particularly when subtle and imperceptible---can be positively applied. For example, motion delay may be leveraged to influence human cognition, such as shaping perceptual judgments or guiding preference formation during observational tasks.”

**Comment 3**

3. figures 1should be contrasting and readable,

**Answer**

Based on this suggestion, we have changed Fig. 1 to ensure it is more contrasting and readable. The details are listed in the response to Comment 1.

**Comment 4**

4. conclusions and future work should be extended to contain practical applications based on research described in this paper - expand references,

**Answer**

Based on this suggestion, we have extended the potential applications of this research in the 5^th^ paragraph of the Discussion section and the second paragraph of the Conclusion section. However, there is a lack of prior research that deliberately applies motion delay as a means to influence human cognition, making it challenging to locate relevant references in the existing literature.

**Discussion section, Paragraph 5 is revised as follows:**

　　“Motion delay is an inherent issue in teleoperational systems. Our research highlights the influence of imperceptible motion delays on preference formation toward computer-generated operator faces when using teleoperated avatars in a virtual environment. One avenue for future work is to test whether this mechanism could also influence the preference formation of operators when observed using a real teleoperated robot. In addition, we can investigate whether other consciousness-related behaviors are also affected by our proposed method. Furthermore, online training systems can leverage motion delays to enhance the learning process by subtly introducing delays when users perform undesired actions, thereby guiding them toward more effective behaviors. Similarly, applications for social interaction support may employ motion delay to indirectly increase the salience or attractiveness of specific objects or individuals. Moreover, as suggested by our results, developers of such systems should not only aim to minimize motion delays as much as possible but also enhance system security to prevent third parties from exploiting these vulnerabilities.”

**Conclusion section, Paragraph 2 is revised as follows:**

　　“Therefore, our findings underscore the negative impact of imperceptible motion delay on preference formation during avatar operation. Apart from shaping preferences, this approach could also influence other cognitive functions, highlighting the broader implications for human--computer interaction and ethical considerations surrounding such technologies. Potential applications include online training systems that introduce motion delays when the operator performs undesirable actions and social interaction support tools that subtly enhance the visual attraction of specific objects. Thus, future studies could explore the impact of imperceptible motion delay on various cognitive functions beyond preference formation, investigate the broader implications for human–computer interaction, address the ethical considerations surrounding the use of such technologies, and focus on developing applications that leverage motion delay in avatar-based systems.”

**Comment 5**

5. if possible, highlight the course of dependencies/relations in figure No. 1 - the yellow color is indistinct,

**Answer**

Based on this suggestion, we have updated Fig. 1. The details are listed in response to Comment 1.

**Comment 6**

6. For ARTICLE type, 19 references are not enough. Please add more references (>19) during your revisions.

I recommend publishing the post after the proposed modifications.

**Answer**

Based on this suggestion, we added four European studies related to our topic. They are “A long distance telesurgical demonstration on robotic surgery phantoms over 5G,” “Virtual reality-based dynamic scene recreation and robot teleoperation for hazardous environments,” “icub3 avatar system: Enabling remote fully immersive embodiment of humanoid robots,” and “Impact of communication delay and temporal sensitivity on perceived workload and teleoperation performance.” Currently, the number of references in this paper is 23.
